# Correlation of retinal fluid and photoreceptor and RPE loss in neovascular AMD by automated quantification, a real‐world FRB! analysis

**DOI:** 10.1111/aos.16799

**Published:** 2024-11-14

**Authors:** Virginia Mares, Gregor S. Reiter, Markus Gumpinger, Oliver Leigang, Hrvoje Bogunovic, Daniel Barthelmes, Marcio B. Nehemy, Ursula Schmidt‐Erfurth

**Affiliations:** ^1^ Laboratory for Ophthalmic Image Analysis, Department of Ophthalmology and Optometry Medical University of Vienna Vienna Austria; ^2^ Department of Ophthalmology Federal University of Minas Gerais Belo Horizonte Brazil; ^3^ RetInSight GmbH Vienna Austria; ^4^ Department of Ophthalmology University Hospital Zurich, University of Zurich Zurich Switzerland

**Keywords:** anti‐VEGF, artificial intelligence, geographic atrophy, image analysis, machine learning, neovascular AMD, optical coherence tomography, photoreceptor, retina

## Abstract

**Purpose:**

To quantify ellipsoid zone (EZ) loss during anti‐VEGF therapy for neovascular age‐related macular degeneration (nAMD) and correlate these findings with nAMD disease activity using artificial intelligence‐based algorithms.

**Methods:**

Spectral domain optical coherence tomography (Spectralis, Heidelberg Engineering) images from nAMD treatment‐naïve patients from the Fight Retinal Blindness! (FRB!) Registry from Zürich, Switzerland were processed at baseline and over 3 years of follow‐up. An approved deep learning algorithm (Fluid Monitor, RetInSight) was used to automatically quantify intraretinal fluid (IRF), subretinal fluid (SRF) and pigment epithelial detachment (PED). An ensemble U‐net deep learning algorithm was used to automated quantify EZ integrity based on EZ layer thickness. The impact of fluid volumes on EZ thickness and late‐stages outcomes were calculated using Wilcoxon rank‐sum tests, a linear mixed model and a longitudinal panel regression model.

**Results:**

Two hundred and eleven eyes from 158 patients were included. The mean ± SD EZ loss area in the central 6 mm was 1.81 ± 2.68 mm^2^ at baseline and reached 6.21 ± 6.15 mm^2^ at month 36. Higher fluid volumes (top 25%) of IRF and PED in the central 1 and 6 mm of the macula were significantly associated with more advanced EZ thinning and loss compared to the low fluid volume subgroup. The high SRF subgroup in the linear regression model showed no statistically significant association with EZ integrity in the central macula; however, the longitudinal analysis revealed an increased EZ thickness with no additional loss.

**Conclusions:**

Intraretinal fluid and PED volumes and their resolution pattern have an impact on alteration of the underlying EZ layer. AI‐supported quantifications are helpful in quantifying early signs of macular atrophy and providing individual risk profiles as a basis for tailored therapies for optimized visual outcomes.

## 1 INTRODUCTION

Optical coherence tomography (OCT) is the gold standard image modality for diagnosing and evaluating disease activity in neovascular age‐related macular degeneration (nAMD) (Schmidt‐Erfurth et al., 2017). Acquiring OCT volumes with high resolution enables a highly accurate identification of biomarkers for retinal diseases including not only fluid volumes, but also precise photoreceptors layers segmentation (Schmidt‐Erfurth & Waldstein, 2016; von der Emde et al., 2023). Each OCT volume contains enormous amounts of data. Thus, artificial intelligence (AI)‐based algorithms working as a tool to analyse many relevant biomarkers on OCT images open a new horizon for a more precise diagnostic and treatment approach in nAMD (Coulibaly et al., 2023; Keenan et al., 2021).

Anti‐angiogenic therapy and in particular, anti‐vascular endothelial growth factor (Anti‐VEGF) are the current standard of care for nAMD (Jaffe et al., 2019). However, better results are more frequently seen early after treatment initiation and some patients may still have poor control of disease activity despite persistent treatment on the long‐term (Nguyen et al., 2019). These findings show the variability in treatment response and in disease activity among different individuals. Moreover, real‐world data compared to clinical trials have a higher variability in treatment outcomes since inclusion/exclusion criteria are less strict. In addition, treatment regimens, as well as retreatment decisions, are dependent on the treating ophthalmologist instead of a strict study protocol (Kiss et al., 2020).

Currently, there is an ongoing discussion about the contribution of intraretinal fluid (IRF), subretinal fluid (SRF) and pigment epithelium detachment (PED) in developing degeneration of the photoreceptors and retinal pigment epithelium (RPE) in nAMD leading to macular atrophy (MA) and/or subretinal fibrosis (SF) (Riedl, Vogl, Mai et al., 2022; Riedl, Vogl, Waldstein et al., 2022; Roberts et al., 2022). It has been postulated that worst functional outcomes in nAMD show a higher correlation with IRF (Riedl, Vogl, Mai et al., 2022; Riedl, Vogl, Waldstein et al., 2022); however, any robust correlation between fluid volumes and visual outcome should contain information about the structural integrity of the neurosensory retina. Previous studies showed a strong and direct correlation between visual acuity and the integrity of outer retinal hyperreflective bands and retinal pigment epithelium (Coscas et al., 2015; Riedl et al., 2020). Long‐term follow‐up is essential to better understand disease progression and poorer outcomes despite anti‐VEGF treatment. The CATT study showed a cumulative proportion of SF increasing from 32% to 56%, and of MA increasing from 12% to 38% from year 1 to year 5 under standardized anti‐VEGF protocols (Daniel et al., 2018; Grunwald et al., 2017). Previous studies showed that photoreceptor damage may be the first step towards RPE degradation and subsequently poor late stage outcomes in AMD (Orlando et al., 2020). Progressive alterations of the ellipsoid zone (EZ) integrity and fluid volumes can now be automated and precisely quantified on OCT. Thus, this study is a proof‐of‐concept of AI‐based EZ integrity quantification and its association with retinal fluid volumes at baseline in real‐world nAMD patients over 3 years of follow‐up.

## 2 METHODS AND MATERIALS

### 2.1 Participant inclusion and grading

This study presents a post hoc analysis of the Fight Retinal Blindness! Registry (FRB!) data including the respective OCT images from a single centre (Zurich, Switzerland). The study was conducted in compliance with the declaration of Helsinki and approval of the respective institutional review boards. Patients with ungradable scans, concomitant retinal sight‐threatening disease and/or presence of MA or SF at baseline, as originally graded by the FRB! investigators, were excluded. Medical records regarding demographic data and best‐correct visual acuity (BCVA) were reviewed. BCVA was measured using Snellen charts and converted to letters on the logarithm of the minimum angle of resolution (logMAR) visual acuity chart. Spectral‐domain (SD) OCT (Spectralis, Heidelberg Engineering) images were processed at baseline and during the 3‐year follow‐up period. The OCT volumes were not standardized; however, the analysed images had to include at least 19 B‐scans per volume. Treatment decisions, including regimen and drug of choice (ranibizumab or aflibercept) had been made by the attending physician. To evaluate the impact of fluid on EZ integrity loss during follow‐up, a high fluid volume subgroup was defined for each compartment (IRF, SRF and PED) including patients with the highest 25% quartile of fluid volume at baseline in the central 6 mm. The remaining 75% of patients were classified as the low fluid volume subgroup for each respective fluid compartment (Schmidt‐Erfurth et al., 2023).

### 2.2 Automated quantification of retinal fluid and EZ segmentation

**FIGURE 1:**
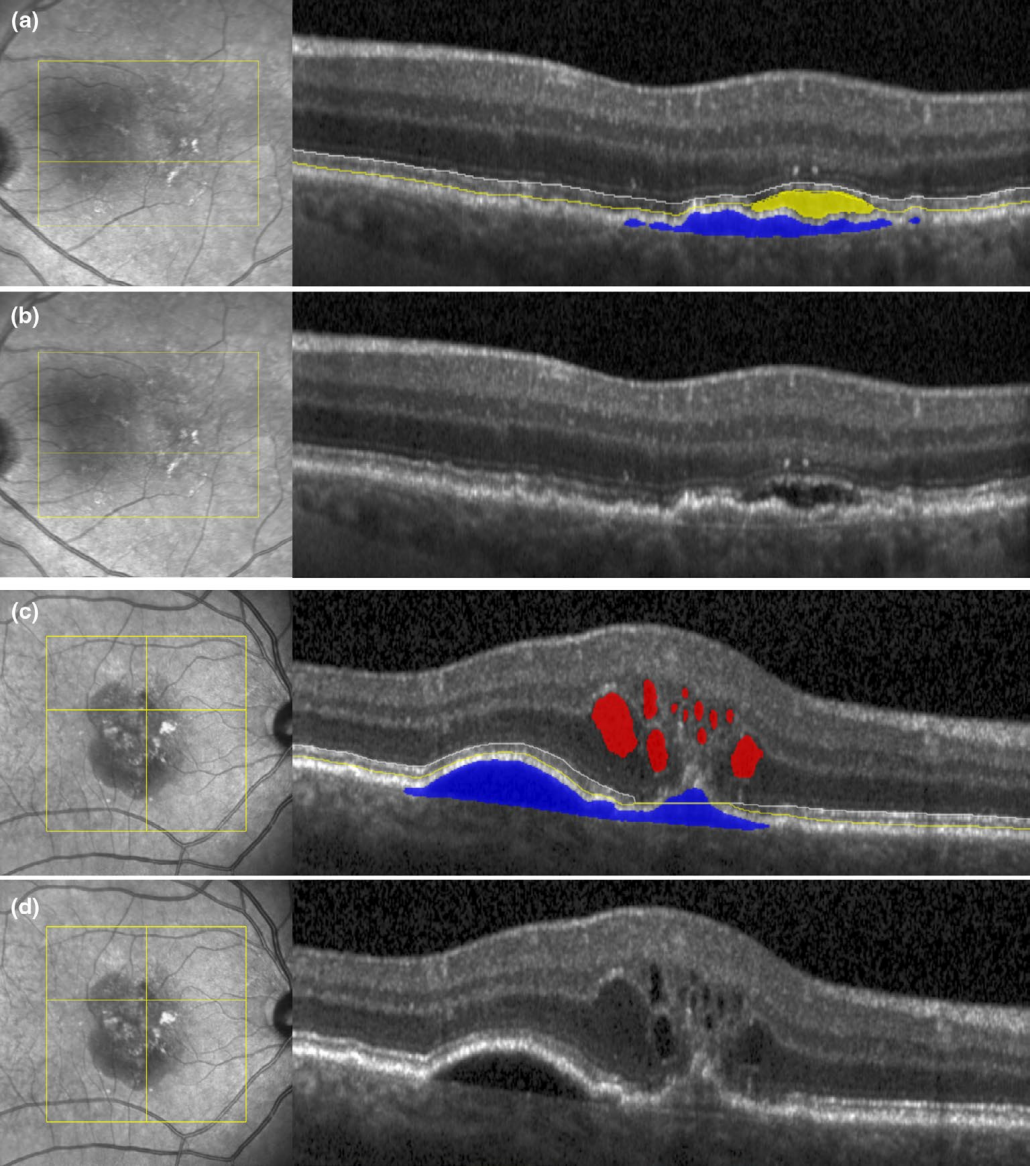
Automated segmentation of intraretinal fluid (red), subretinal fluid (yellow) and pigment epithelial detachment (blue), and ellipsoid zone (EZ) thickness. EZ thickness is reliably segmented when the fluid volume is below the threshold of 60 nL in the central 6 mm.

Macular fluid was automatically segmented and quantified using an extensively validated AI algorithm (Fluid Monitor, RetInSight) (Schlegl et al., 2018). The algorithm is approved by the medical device regulation (MDR) (EU) 2017/745 and uses a convolutional neural network (CNN) to identify retinal fluid on a pixel‐level in each compartment (Schlegl et al., 2018). Absolute volume quantities were computed in nanolitres (nL) (1 nL = 0.001 mm^3^) within the central 1 and 6 mm macular fields. PED was defined as a segmented region between the retinal pigment epithelium (RPE) and Bruch's membrane with a height > 200 μm, or alternatively, a width > 400 μm, as originally defined by professional reading centres (Vienna, Wisconsin, Duke) (Schmidt‐Erfurth et al., 2023). Figure 1 shows a segmentation of PED. EZ integrity was defined as continued segmentation of the area between the top of the EZ and the outer boundary of the interdigitation zone. Automated segmentation of EZ thickness was performed by a previously reported ensemble of U‐Net‐based fully CNN delineating layers on each individual B‐scan of the entire SD‐OCT volume (Orlando et al., 2020; Riedl, Vogl, Mai et al., 2022; Riedl, Vogl, Waldstein et al., 2022).

From the voxel‐level binary segmentation, an en‐face EZ thickness map was calculated, providing a thickness value for each single A‐scan. In a postprocessing step, EZ integrity loss was defined as a discontinuity of the previously described segmented layer. For the automated EZ quantification, OCT scans with fluid volume over 60 nL in the central 6 mm area or over 10 nL in the central 1 mm area were excluded. Figure 1 shows examples of segmentation with less than 60 nL of fluid in the macula. High fluid volumes will distort adjacent structures to a level that the EZ layer cannot be reliably segmented on OCT. The following time points were used in the analysis: month 3 (M3), M6, M12, M15, M24, M27 and M36. Spatiotemporal correlation of fluid volumes in nL with EZ loss in mm^2^ and μm was performed to identify early signs of atrophy development and progression in nAMD in dependence on the fluid compartments and their respective volume.

### 2.3 Statistics and mathematical analysis

Data were split between high (top 25%) and low (bottom 75%) of the macular fluid volumes groups in each compartment at baseline. A comparison between high versus low macular fluid volumes impact on EZ loss was performed using a Wilcoxon rank‐sum tests with bootstrapped confidence intervals. EZ thickness and integrity loss and their association with the development of MA and/or SF were calculated using a linear mixed model in which the fixed effects were the time points and development of MA or SF within the 36 months. The random effect in these calculations was patient (eye). A longitudinal panel regression model was also performed to correlate fluid volumes to EZ thickness and EZ loss. For all statistical tests, a *p*‐value below the significance level 0.05 was considered significant.

## 3 RESULTS

Four hundred and thirty eyes from 215 treatment‐naïve patients were initially evaluated. Ungradable scans, and eyes with other concomitant macular disease and/or presence of geographic atrophy or fibrosis at baseline were excluded. The final sample consisted of 211 eyes from 158 patients with nAMD. The mean number of injections were 18.57 ± 8.74 over the 3 years of follow‐up.

### 3.1 EZ thickness and EZ integrity loss measurements

There was a progressive increase in the EZ loss area during follow‐up. In the total cohort, the mean ± SD of EZ loss area in the central 6 mm at baseline was 1.81 ± 2.68 mm^2^, increased to 4.88 ± 5.4 mm^2^ after initial treatment (month 3), reached 5.19 ± 5.43 mm^2^ at month 24 and 6.21 ± 6.15 mm^2^ at month 36.

There was a continuous thinning of the EZ layer (measured from top of the ellipsoid zone to the outer boundary of the interdigitation zone) during follow‐up. The mean EZ thickness in the central 6 mm at baseline was 26.9 ± 4.7 μm, decreased to 22.71 ± 5.77 μm at month 3, reaching 22.04 ± 5.38 μm at month 24 and 21.4 ± 5.8 μm at month 36 as shown in Table 1. No significant change in EZ integrity loss area was detected in the central 1 mm from baseline to month 36.

**TABLE 1:** Ellipsoid zone loss, EZ thickness, subretinal fluid, intraretinal fluid and pigment epithelium detachment changing at baseline and months 3, 6, 12, 24 and 36.

	BSL 6 mm	M3 6mm	M6 6mm	M12 6 mm	M24 6 mm	M36 6 mm
EZ loss (μm^2^)	1.81 ± 2.68	4.88 ± 5.4	4.56 ± 5.43	4.21 ± 4.45	5.19 ± 5.43	6.21 ± 6.15
Mean EZ thickness (μm)	26.9 ± 4.76	22.71 ± 5.77	22.71 ± 5.43	22.74 ± 4.96	22.04 ± 5.38	21.45 ± 5.82
Median SRF (nL)	133.38 (IQR 7.79–441.71)	0.99 (IQR 0.05–17.18)	3.89 (IQR 0.13–23.75)	3.64 (IQR 0.33–30.8)	1.5 (IQR 0.0–14.95)	1.29 (IQR 0.17–16.87)
Median IRF (nL)	1.18 (IQR 0.0–80.5)	0.0 (IQR 0.0–1.83)	0.0 (IQR 0.0–5.06)	0.13 (IQR 0.0–5.43)	0.0 (IQR 0.0–23.28)	0.29 (IQR 0.0–8.05)
Median PED (nL)	0.0 (IQR 0.0–720.27)	0.0 (IQR 0.0–405.31)	0.0 (IQR 0.0–755.04)	0.0 (IQR 0.0–503.11)	0.0 (IQR 0.0–678.3)	0.0 (IQR 0.0–750.66)

Abbreviations: BSL, baseline; EZ, ellipsoid zone; IRF, intraretinal fluid; M, month; nL, nanolitre; PED, pigment epithelium detachment; SRF, subretinal fluid; μm, micometre.

**FIGURE 2:**
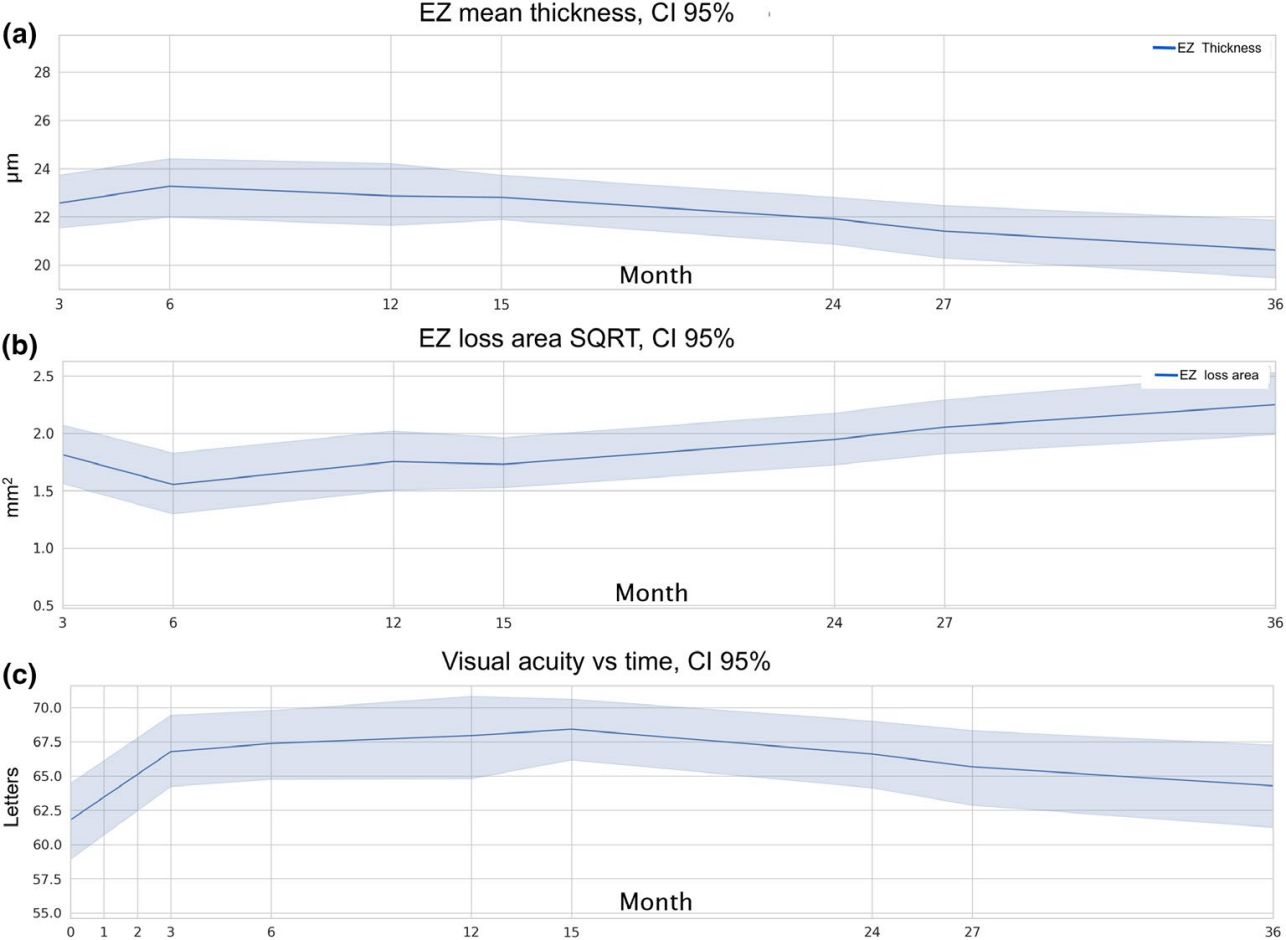
The plots show a continuous thinning on the ellipsoid zone (EZ) (a), a progressive increase in the EZ loss area (b) and a decrease in visual acuity particularly after month 15 (c).

Overall, the continuous thinning on the EZ layer was followed by a progressive loss of its integrity from month 6 until month 36 (Figure 2). Related visual acuity showed more impressive improvement from baseline to month 3, and then maintained a modest improvement until month 15. Thereafter, a progressive worsening on visual acuity was noted (Figure 2). The Spearman correlation between EZ loss and visual acuity at month 36 was moderate to high with −0.61 (*p* < 0.0001).

### 3.2 Fluid correlation with EZ affection

**FIGURE 3:**
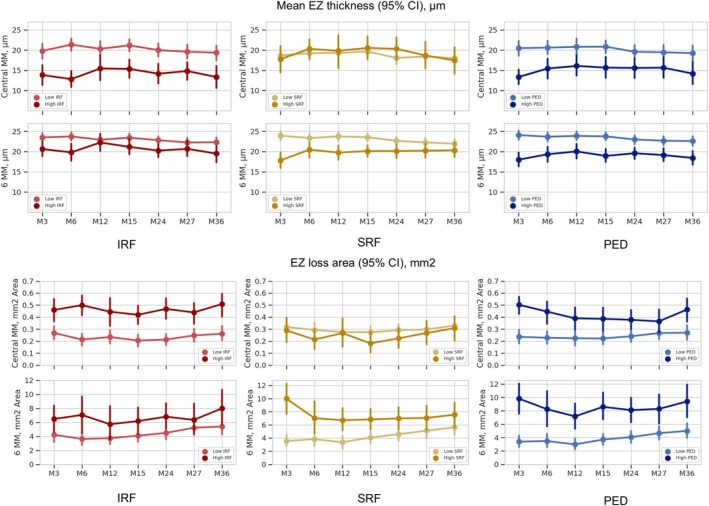
A comparison between higher fluid volume subgroup (top 25%) in each compartment (intraretinal and subretinal fluid and pigment epithelial detachment) and bottom 75% fluid volume subgroup regarding ellipsoid zone (EZ) thickness and EZ loss during follow‐up.

At baseline, SRF was present in 87.68% and IRF was present in 60.19% of all scans. The median volumes of SRF and IRF at baseline in the central 6 mm were 133.38 nL (interquartile range [IQR]: 7.79–441.71) and 1.18 nL (IQR: 0–80.5), respectively. Higher fluid volume subgroup (top 25%) of IRF in the central 1 and 6 mm of the macula were significantly associated with more EZ thinning in the analysed time points (*p* = 0.001, 95%CI 2.51–8.85 and *p* = 0.01 95%CI 0.44–5.17 at month 36, respectively). Higher volume of PED in the central 1 and 6 mm also showed significant association with EZ thinning in the analysed time points (*p* = 0.003, 95% CI 2.12–8.85, *p* = 0.001, 95% CI 2.14–6.27, respectively, at month 36). Higher IRF volume subgroup also showed a significant correlation with the EZ loss area in the central 1 mm (*p* = 0.0002, 95% CI −5.45 to −1.96 at month 36). However, after Bonferroni correction, this correlation was not significant in the central 6 mm. High PED volumes in the central 6 mm were significantly correlated with EZ loss in the analysed time points (*p* = 0.0001, 95% CI −5.46 to −2.25 at month 36). In contrast, high volume of SRF showed no statistically significant association with EZ thinning in the central 1 and 6 mm or EZ loss in the central 1 mm during follow‐up (*p* = 0.94, 95% CI −3.40 to 3.89; *p* = 0.18, 95% CI −0.49 to 3.84; and *p* = 0.64, 95% CI −1.47 to 2.40, respectively, at month 36). These correlations are shown in Figure 3.

A longitudinal panel analysis confirmed a significant correlation between PED volume and EZ thinning (*p* = 0.01), as well as with an increase in the area of EZ loss within the central 6 mm (*p* = 0.004). IRF showed a clear correlation with EZ thinning in the central 1 mm (p = 0.01), which was not statistically significant in the central 6 mm, but still showed a trend towards a thinning effect (*p* = 0.05). The difference may be due to the fact that most of the IRF is present within the central 1 mm and the effect may be less when the entire 6 mm area is included. Regarding EZ loss, IRF volume showed no significant correlation with EZ loss in the central 1 and 6 mm. SRF was significantly correlated with an increase in EZ thickness and showed a negative correlation with the area of EZ loss (*p* < 0.001 and *p* = 0.002, respectively).

### 3.3 EZ loss with development of macular atrophy (MA) and subretinal fibrosis (SF)

Despite regular treatment in clinical practice, 34.1% (72 eyes) converted to late stages of the disease (MA and SF) over 3 years of follow‐up. Thirty‐six eyes developed MA, 10 eyes developed SF and 26 eyes developed combined MA and SF. When analysing the impact of EZ behaviour during the late‐stage outcomes in this cohort, we found that eyes which developed MA and SF (converters to late‐stage of nAMD) had a significant correlation with faster progression of the EZ loss area (steeper curve slope) in the central 1 and 6 mm (*p* = 0.01 and *p* = 0.001, respectively). Furthermore, EZ thinning in the central 1 mm was significantly correlated with MA and SF development (*p* = 0.04), but this was not seen on the central 6 mm (*p* = 0.50).

## 4 DISCUSSION

High‐resolution optical coherence tomography (OCT) is a non‐invasive imaging modality with close correlation with retinal histology (Cheng et al., 2018). The detailed analysis of OCT scans may provide an accessible opportunity to analyse the behaviour of the neurosensory layers in vivo during AMD progression. As observed in both trials and clinical practice, late‐stage outcomes in nAMD involving photoreceptor damage persist despite continued treatment with anti‐VEGF. The presence of fluid remains a significant contributing factor in these outcomes. Our overall results showed a progressive decrease in EZ thickness and a progression in EZ loss in close correlation with visual acuity changes after 6 months and thereafter (Figure 2). Although macular atrophy origins remain unclear, photoreceptor integrity loss is clearly a key feature in the early stage of atrophy development, and therefore, an important biomarker of disease progression (Riedl, Vogl, Mai et al., 2022; Riedl, Vogl, Waldstein et al., 2022). The effect of ageing on the retinal tissue is heterogeneous and closely related with areas of higher energy demands. Thus, the macula and specially the outer retina and choroid are major targets in age‐related retinal diseases (Elsner et al., 2022; Zouache, 2022). Photoreceptor and posterior RPE degradation with development of macular atrophy is important for long‐term results of visual acuity in non‐neovascular AMD as well as in nAMD.

In this cohort, SRF was the most prevalent fluid component at baseline and 87.68% of all scans had SRF while 60.19% had IRF in the central 6 mm. These rates are similar to related data from clinical studies with even larger populations such as the HARBOR trial (Sadda et al., 2022). The larger amount of total retinal fluid present at baseline can cause distortions in the outer retinal bands as well as transitory worsening of visual acuity. Therefore, the quantification of EZ integrity loss at baseline and its correlation with visual acuity may be not reliable at this time point. Yet, a follow‐up quantification of EZ thickness and the comparison between the higher and lower fluid volume subgroups can be more reliably performed from month 3 to month 36.

The direct correlation between retinal fluid volumes and visual acuity may be unreliable, due to varying pre‐existing damage to the underlying neurosensory compartment. However, EZ loss and visual acuity are closely related which was demonstrated in this study. A progressive EZ loss followed by a continuous decrease in visual acuity after month 15 was observed. Therefore, photoreceptor layer analysis on OCT can be used not only as a biomarker in intermediate AMD (Zekavat et al., 2022) and GA, but also in nAMD. Furthermore, a binary classification of presence or absence of ellipsoid zone does not completely meet the actual interest in automated quantification of outer retinal bands on OCT.

Photoreceptor thinning has been described preceding their complete loss in histological studies, nevertheless the pathophysiology remains unclear (McHugh et al., 2019; Riedl, Vogl, Mai et al., 2022; Riedl, Vogl, Waldstein et al., 2022). Yet, photoreceptor thinning has also been correlated with loss of visual function using microperimetry measurements in drusen areas (Acton et al., 2012). The possibility of real‐time correlation of retinal fluid behaviour and neurosensory disease progression is clearly providing a missing link in the understanding of fluid/function correlation. In real world, automated segmentation is mandatory, since manually segmentation and quantification of retinal layers and fluid volumes are time consuming and not realistic in clinical routine.

Currently, automated tools such as central retinal thickness (CRT) have been used in clinical practice as a marker of disease activity or recurrence and to guide treatment decisions in nAMD. However, this measurement does not provide any detailed information about location and extension of disease‐specific activity, and also presented a weak correlation with visual acuity and fluid volumes (Nanegrungsunk et al., 2022; Pawloff et al., 2022). Previous studies have postulated that the severity of CRT fluctuation is related with visual acuity outcomes (Chakravarthy et al., 2021; Lai and Lai, 2021; Sheth et al., 2022); thus, an automated tool capable of measuring and analysing fluid fluctuation in each compartment for each patient during follow‐up may provide more accurate and personalized information about functional and anatomical outcomes. The comparison between high and low fluid volume subgroups showed that the central macula is particularly vulnerable to damage caused by high IRF volumes, leading to EZ thinning and loss. This is in accordance with previous analysis in the FRB! Registry dataset that indicates higher IRF volumes associated with increased atrophy onset (Nguyen et al., 2021). Since photoreceptor damage may be the first step to RPE degradation and persistent macular atrophy (Orlando et al., 2020), this analysis becomes crucial. The longitudinal analysis confirmed statistically significant correlation between IRF and EZ thinning in the central 1 mm. In the central 6 mm area, *p* = 0.05, indicating a trend that did not reach statistical significance. Regarding IRF and EZ loss correlation, the longitudinal analysis showed no statistically significant correlation. Since the IRF volumes are mainly located in the central macula, both models presented more significant damage in the 1 mm area.

The subgroups analysis showed that high SRF volumes were neither significantly associated with EZ thinning in the central 1 or 6 mm, nor with EZ loss. Interestingly, the longitudinal panel analysis suggests that SRF correlates with an increase of EZ thickness and presented a negative correlation with EZ loss. Supporting this finding, previous analysis have shown that inactive persistent SRF volumes may be tolerated and may also be related to better visual outcomes (Bogunović et al., 2022; Guymer et al., 2019). However, when the residual volume of SRF is higher and actively increases, it does lead to worse visual acuity outcomes (Grechenig et al., 2021; Sadda et al., 2022). Furthermore, there might be an increase in the number of injections needed to achieve disease control in the first year of follow‐up (Bogunović et al., 2022; Mares et al., 2023). Higher PED volumes in the central 1 and 6 mm of the macula were also significantly associated with EZ thinning and integrity loss during follow‐up. Schuman et al. (2009) have demonstrated an association of large drusen with the loss of photoreceptor and outer segments in 2009 and so, it is expected that in larger PEDs that would also be noted.

The relentless progression of photoreceptor degeneration in nAMD despite regular treatment remains a clinical unmet need. In previous studies, IRF has been linked to deteriorating visual acuity outcomes and the onset of atrophy (Reiter et al., 2023; Riedl, Vogl, Mai et al., 2022; Riedl, Vogl, Waldstein et al., 2022). Our analysis comparing EZ behaviour on eyes that developed late stages of nAMD such as atrophy and fibrosis, during a 3‐year follow‐up with those that did not, supported previous findings in the literature. A faster increase of the EZ loss area in both the central 1 mm and 6 mm area were found to be significantly associated with further development of clinical signs of atrophy and/or fibrosis. Thus, understanding the impact of retinal fluid volumes on the outer retina segments may be the key to better understand and maybe prevent late‐stage outcomes of nAMD. In times of the first pharmacological treatments for GA being approved, combination therapies may be an option, although having to take into account MNV formation under GA therapy.

A possible association of fluid compartment, MNV type and late‐stage outcomes in nAMD has been suggested in the literature (Ernest et al., 2020). Type 3 MNV has been more frequently associated with IRF and macular atrophy. Type I MNV showed predominantly SRF and a protective effect for macular atrophy has been postulated (Fukuyama et al., 2022). In our analysis, MNV type showed no significant correlation with increased EZ thinning and/or loss during follow‐up. However, type 3 MNV was presented in only six cases, and this must be noted as a limitation, thus no final conclusions can be taken from such a small subgroup.

This is a retrospective real‐world study and therefore has some inherent limitations such as retrospective chart analysis, non‐standardized treatment decisions and different scan mode imaging between different patients and within patients and between all visits.

However, this is a large sample size of treatment‐naïve patients, from the well‐curated FRB! Registry platform, which offers high‐quality data from routine disease management (Gillies et al., 2014). Furthermore, OCT images were collected from one single device (OCT Spectralis, Heidelberg Engineering) avoiding technical variability among device types. Yet, the AI algorithms used in the analysis were previously tested, human ground truth was provided and the tools were validated. EZ automated segmentation was only possible in the absence of large fluid volumes or with low amounts of fluid at the defined timepoints (less than 60 nL in the 6 mm area, as described in the methods). Neovascular AMD comes with the dilemma of a fast, devastating and usually irreversible loss of retinal function without treatment (Kiss et al., 2020). Retinal fluid particularly intraretinally may cause permanent damage to the EZ in the central macula with resulting irreversible visual loss. Interesting is also the notion that PED is associated with a decline of EZ integrity. As demonstrated in this study, volumes and location of retinal fluid compartments are important for neurosensory layer anatomy and, consequently, for visual outcomes. Prompt detection of photoreceptor early changes associated with fluid dynamics may be the most impactful method to optimize anti‐VEGF treatment and try to prevent long‐term visual loss due to nAMD. Advanced OCT image analysis shed more light into the complex patterns of nAMD and AMD in general. A precise quantitative analysis of fluid by type and volume in nAMD and knowing about the pathognomonic impact of fluid on EZ and RPE as correlate for visual function may empower health care providers to develop a comprehensive approach to vison‐saving therapy. The portfolio of relevant biomarkers and parameters guiding therapeutic strategies is increasing as we speak and is ready to be used in the hands of thousands of ophthalmologists treating millions of patients which are threatened by a blinding disease. In times of AI‐based precision measurements, fluid volumes and neurosensory destruction can be visualized in every individual patient to improve his/her outcome and throughout populations to understand the associations of markers of nAMD and AMD disease in general. Macula‐wide EZ integrity assessment work is certainly a most useful candidate endpoint to monitor nAMD treatment and progression.

## FUNDING INFORMATION

This study received no funding.

## CONFLICT OF INTEREST STATEMENT

GSR: Research funds from RetInSight. Consultant for Apellis, Bayer, Boehringer Ingelheim and Roche. HB: Research funds from Heidelberg Engineering and Apellis. DB: Research grants and travel expenses from Bayer and Novartis. (b) Scientific consultant for Alcon. US‐E: Scientific consultant for AbbVie, Annexon, Apellis, Aviceda, Complement Therapeutix, Genentech, Heidelberg Engineering, Kodiak, RetInSight, Novartis, Roche and Topcon. OL: Employee of RetInSight. VM, MG and MBN report nothing to declare.
